# Genetic Model to Study the Co-Morbid Phenotypes of Increased Alcohol Intake and Prior Stress-Induced Enhanced Fear Memory

**DOI:** 10.3389/fgene.2018.00566

**Published:** 2018-11-27

**Authors:** Patrick Henry Lim, Guang Shi, Tengfei Wang, Sophia T. Jenz, Megan K. Mulligan, Eva E. Redei, Hao Chen

**Affiliations:** ^1^Department of Psychiatry and Behavioral Science, Feinberg School of Medicine, Northwestern University, Chicago, IL, United States; ^2^Liaoning Provincial People’s Hospital, Liaoning Sheng, China; ^3^Department of Pharmacology, University of Tennessee Health Science Center, Memphis, TN, United States; ^4^Department of Genetics Genomics and Informatics, University of Tennessee Health Science Center, Memphis, TN, United States

**Keywords:** alcohol self-administration, genetic model of depression, contextual fear conditioning, corticosterone, glucocorticoid receptor, alcohol use disorder, post traumatic stress disorder, inbred rat strains

## Abstract

Posttraumatic Stress Disorder (PTSD) is a complex illness, frequently co-morbid with depression, caused by both genetics, and the environment. Alcohol Use Disorder (AUD), which also co-occurs with depression, is often co-morbid with PTSD. To date, very few genes have been identified for PTSD and even less for PTSD comorbidity with AUD, likely because of the phenotypic heterogeneity seen in humans, combined with each gene playing a relatively small role in disease predisposition. In the current study, we investigated whether a genetic model of depression-like behavior, further developed from the depression model Wistar Kyoto (WKY) rat, is a suitable vehicle to uncover the genetics of co-morbidity between PTSD and AUD. The by-now inbred WKY More Immobile (WMI) and the WKY Less Immobile (WLI) rats were generated from the WKY via bidirectional selective breeding using the forced swim test, a measure of despair-like behavior, as the functional selector. The colonies of the WMIs that show despair-like behavior and the control strain showing less or no despair-like behavior, the WLI, are maintained with strict inbreeding over 40 generations to date. WMIs of both sexes intrinsically self-administer more alcohol than WLIs. Alcohol self-administration is increased in the WMIs without sucrose fading, water deprivation or any prior stress, mimicking the increased voluntary alcohol-consumption of subjects with AUD. Prior Stress-Enhanced Fear Learning (SEFL) is a model of PTSD. WMI males, but not females, show increased SEFL after acute restraint stress in the context-dependent fear conditioning paradigm, a sexually dimorphic pattern similar to human data. Plasma corticosterone differences between stressed and not-stressed WLI and WMI male and female animals immediately prior to fear conditioning predict SEFL results. These data demonstrate that the WMI male and its genetically close, but behaviorally divergent control the WLI male, would be suitable for investigating the underlying genetic basis of comorbidity between SEFL and alcohol self-administration.

## Introduction

Comorbid posttraumatic stress disorder (PTSD) and alcohol use disorder (AUD) is a prevalent and devastating disorder. While exposure to a traumatic event is required for diagnosis, not all subjects who experience a traumatic event develop PTSD. PTSD has an overall lifetime prevalence rate of 7–8% and is the fifth most common major psychiatric disorder in the United States (Keane et al., 2006). PTSD has a high comorbidity with major depression (50–84%; Spinhoven et al., 2014; Flory and Yehuda, 2015) and other anxiety disorders (49%; Smith et al., 2016). AUD occurs in 30% of PTSD subjects and up to 54% in veterans (Smith et al., 2016). The cause of these high comorbidities might be that the preexistence of these disorders increases susceptibility to traumatic events (Breslau, 2009) or that all these disorders are the consequences of the traumatic exposure (Pietrzak et al., 2011). However, the odds of having PTSD are 30% greater for those with a lifetime AUD (Grant et al., 2015, 2016). Individual differences in heritable factors affect the risk to develop PTSD. Twin studies show that the heritability of PTSD alone is between 30–46% (Stein et al., 2002; Wolf et al., 2014). Nevertheless, genome-wide association studies have had limited success identifying these factors (Duncan et al., 2018; Polimanti et al., 2018), which is not surprising given the heterogeneity of the symptoms and comorbidities. Individual variations in comorbidity, namely that some PTSD patients do, while others do not have AUD, supports the hypothesis of overlapping genetic vulnerability to PTSD and AUD in some patients (McLeod et al., 2001; Frías and Palma, 2015). Critically, there is a paucity of data on the genetic vulnerabilities that cause the PTSD-AUD comorbidity. Novel animal models may lead to the identification of genetic vulnerabilities to this comorbidity and potentially to effective treatments for this refractory condition. Comorbidity with a disorder or illness is commonly defined as a condition existing simultaneously with and usually independently of another medical condition. The ideal animal model of comorbidity thus would show intrinsic characteristics modeling both conditions. One of the goals of this study is to establish a rat model of comorbidity between PTSD and AUD.

Valid animal models could help identify the genetic components of PTSD vulnerability. Among the many proposed rodent models, consensus as to what constitutes a PTSD-like model is just starting to emerge. PTSD has been related to exaggerated implicit fear memory, resulting from associative fear conditioning and non-associative sensitization processes (e.g., Foa et al., 1992; Charney et al., 1993). Most models have in common the study of emotional memories according to a Pavlovian learning paradigm. This associative learning process consists of the pairing of a neutral conditioned stimulus, such as a place or context, with an aversive unconditioned stimulus (e.g., shock) eliciting a conditioned fear response. Prior stress enhances fear conditioning in male rodents with long-term consequences (Blouin et al., 2016). This model represents an environmental enhancement (by prior stress) of fear memory, which closely reproduces many core symptoms of PTSD, including enhanced fear learning, generalized anxiety and impaired extinction (Rau et al., 2005).

Pavlovian fear conditioning also mimics the traumatic event-induced symptoms of intense and recurrent fear, characteristic of patients with PTSD (Zovkic and Sweatt, 2013). The advantages of fear conditioning as a model of PTSD include: (1) stress-induced exaggeration of fear conditioning, induced by exposing rodents to trauma prior to fear conditioning, models the environment-induced predisposition to PTSD; (2) rodent strains that vary in stress-induced exaggeration of fear memory can be used to dissect genetic predisposition to PTSD; (3) brain regions involved in the regulation of fear conditioning have also been implicated in the psychopathology of PTSD (Bremner, 2007; Hall et al., 2012; Zoladz and Diamond, 2013), and (4) the measurement of fear memory is automated and highly reproducible.

Stress-enhanced fear learning (SEFL) is an animal model of PTSD that encompasses both stress-sensitizing effects and conditioned fear memory components of the PTSD pathology (Rau et al., 2005; Blouin et al., 2016). Stress-induced release of peripheral corticosterone (CORT) has been suggested to be necessary for SEFL induction, and these changes are mediated by glucocorticoid (Nr3c1) and mineralcorticoid (Nr3c2) receptors (Donley et al., 2005; Rodrigues et al., 2009; Perusini et al., 2016). If prior stress exaggerates fear memory in one strain of animals, but not in another, it may present a model of PTSD that mirrors the variability in susceptibility to PTSD in humans (Skelton et al., 2012). Through a genetic animal model of depression, we aim to investigate the comorbid relationship between increased alcohol intake, acute stress-induced changes in fear memory and possible sex differences in these measures.

The genetic rat model of depression was developed from the near-inbred Wistar Kyoto (WKY) rat strain, an established model of major depression with co-morbid anxiety (Pare and Redei, 1993a,b; Pare, 1994; Solberg et al., 2001, 2004; Baum et al., 2006) and hormonal and sleep characteristics similar to those of depressed humans. Two inbred strains were generated from the WKYs by selective breeding based on immobility behavior in the forced swim test, resulting in two nearly isogenic inbred strains of their 38–41th generation at the time of this study. These are the WKY More Immobile (depressed WMI) showing despair-like behavior and its genetically similar, but behaviorally different control strain, the Wistar Kyoto Less Immobile (WLI). The WMI rats consistently display depression-like behavior in the forced swim test compared to their genetically very close less immobile (WLI) control strain (Will et al., 2003; Andrus et al., 2012; Mehta et al., 2013).

We hypothesized that WMIs would likely display increased fear memory sensitized by a prior acute stress, as 50–84% of PTSD patients have been diagnosed with major depression as well. We further hypothesized that WMI would likely consume more alcohol than the WLI controls based on clinical reports that the odds of having PTSD are 30% greater for those with AUD and major depression is also co-morbid with AUD. To test these hypotheses and the possibility that WMIs could serve as genetic model for the comorbidity of PTSD and AUD, we exposed adult male and female WMIs and WLIs to SEFL and another set of animals to an operant alcohol drinking paradigm.

## Materials and Methods

### Experimental Design and Statistical Analysis

Different cohorts of animals were used in the three different sets of experiments. In the first set of experiments, 6 to 7-month-old WLI and WMI males and females were either exposed to acute restraint stress (ARS) or received no stress (NRS). Forty-eight hours later they were exposed to contextual fear conditioning (CFC). In the second set of experiments, age matched male and female rats were exposed to ARS or NRS and then sacrificed 48 h later without CFC test to measure brain gene expression and plasma corticosterone levels. The third set of WLI and WMI male and female animals received alcohol via a self-administration protocol. Sample numbers are indicated in the figure legends.

Activity and fear memory in the CFC, and plasma corticosterone levels were analyzed first by three-way ANOVA (strain, sex, and stress), followed by separate two-way ANOVAs for males and females to identify strain and stress effects, specifically. Bonferroni corrections for *post-hoc* comparisons were used to identify differences between groups. ANOVA results are described in the results section, while *post-hoc* comparisons are indicated on the figures and in the figure legends. Significance was considered *p* < 0.05. Effect size (Cohen’s d; Cohen, 1988) was calculated using the ‘effsize’ package of the R language. Statistical analyses were carried out by GraphPad Prism 7 (La Jolla, CA, United States) and Systat softwares.

### Animals and Behavioral Tests

The animal colonies are maintained at Northwestern University, Feinberg School of Medicine and at the University of Tennessee Health Science Center. The Institutional Animal Care and Use Committee of Northwestern University and the University of Tennessee Health Science Center approved all animal procedures. The guidelines described in the Public Health Service Policy on Humane Care and Use of Laboratory Animals are followed. Animals were housed in a temperature and humidity-controlled environment. Male and female WMIs and WLIs were employed at 6 to 7-months-of-age.

#### Acute Restraint Stress

The rats were placed into flexible plastic bags with an opening for their mouth and nose. The animals could not turn around in this apparatus nor move the plastic bags. The restraint was conducted between 1200–1600 h. After 2 h, the rats were placed back into their home-cages and 48 h later, one cohort was sacrificed by fast decapitation to obtain blood and brain tissue and another cohort was tested in contextual fear conditioning.

#### Contextual Fear Conditioning

Rats were placed into an automated fear conditioning apparatus of Technical & Scientific Equipment (TSE, Bad Homburg, Germany) for 3 min of habituation, followed by three mild shocks (0.8 mA, 1 s per min) over 3 min. Between animals, the chamber was cleaned using 75% ethanol to eliminate behavioral changes caused by odor. Twenty-four hours later, the rats were placed in the same chamber for 3 min without any shocks and examined for contextual fear memory as measured by freezing duration and total locomotion (distance traveled) through the use of an infrared beam system (detection rate 10 Hz). Any rats that did not respond to the initial shock were excluded from the study.

#### Operant Alcohol Self-Administration

**FIGURE 1:**
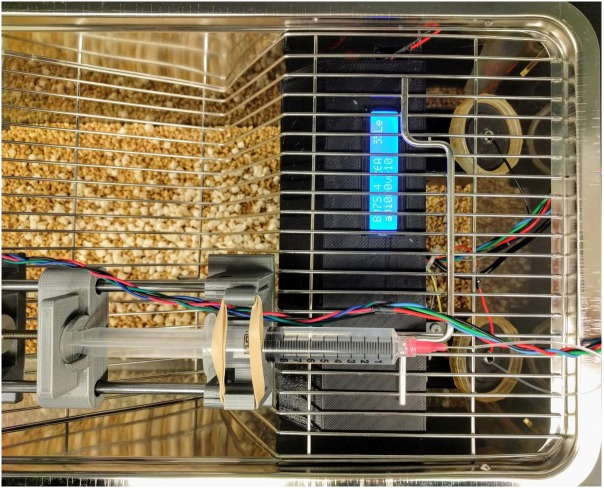
Operant licking device for oral alcohol self-administration. The device has a lick sensor that records licking events from both the active and inactive spouts. Licking on the active spout meeting a variable ratio schedule triggers the syringe pump to push one drop (60 μl) of alcohol to the tip of the spout. A visual cue (an LED) is turned on for 1 s with each reward. Licking on the inactive spout has no programed consequence.

Oral alcohol self-administration using licking as the operant response was carried out in an open source device we described previously (Longley et al., 2017). Figure 1 shows the device employed for alcohol self-administration. It is constructed from a 3D printed frame and contained a single-board computer that monitors two licking spouts. When the number of licks on the active spout met variable ratio 10 reinforcement criteria, the computer advanced a syringe pump and pushes one drop (60 μl) of 6.5% alcohol to the tip of the spout. A visual cue (an LED) was turned on for 1 s with each reward. Licking events were recorded on the inactive spout, or on the active spout within the 20 s timeout period after the reward was delivered but no consequence are programmed for these events. Rats were tested individually during the light off phase of the diurnal cycle without prior operant training, water deprivation or sucrose training. A total of 10 daily 1 h sessions were conducted.

### Plasma Corticosterone (CORT) ELISA

Trunk blood samples were collected into EDTA-coated tubes on ice. The samples were centrifuged at 3500 rpm for 10 min. Plasma was stored at -80°C for later analysis. Plasma corticosterone levels were measured by immunoassay using a commercially available competitive ELISA kit (Corticosterone Competitive ELISA kit, ThermoFisher, United States).

### Measurement of Hippocampal Glucocorticoid and Mineralocorticoid Receptor mRNA Levels

Rat brains were rapidly dissected on ice using Paxinos coordinates (Paxinos and Watson, 2013) for the whole hippocampus (AP-2.12 to -6.0, ML 0 to 5.0, DV 5.4 to 7.6). Tissues were collected into RNAlater reagent (Ambion, Austin, TX, United States) and stored at -80°C.

RNA extraction using the Direct-zol RNA Miniprep kit (Zymo Research, Irvine, CA, United States) and quantitative PCR (qPCR) using the QuantStudio 7 Flex Real-Time PCR System (Thermo Fisher Scientific, Waltham, MA, United States) were performed as described previously (Tunc-Ozcan et al., 2017). Target gene expression (*Nr3c1* and *Nr3c2*) was normalized to *Gapdh* as the housekeeping gene and a general calibrator using the 2^-ΔΔCt^ method. Primer sequences are listed in Supplementary Table 1.

## Results

### Stress Enhanced Fear Learning

#### Habituation to Test Environment

**FIGURE 2:**
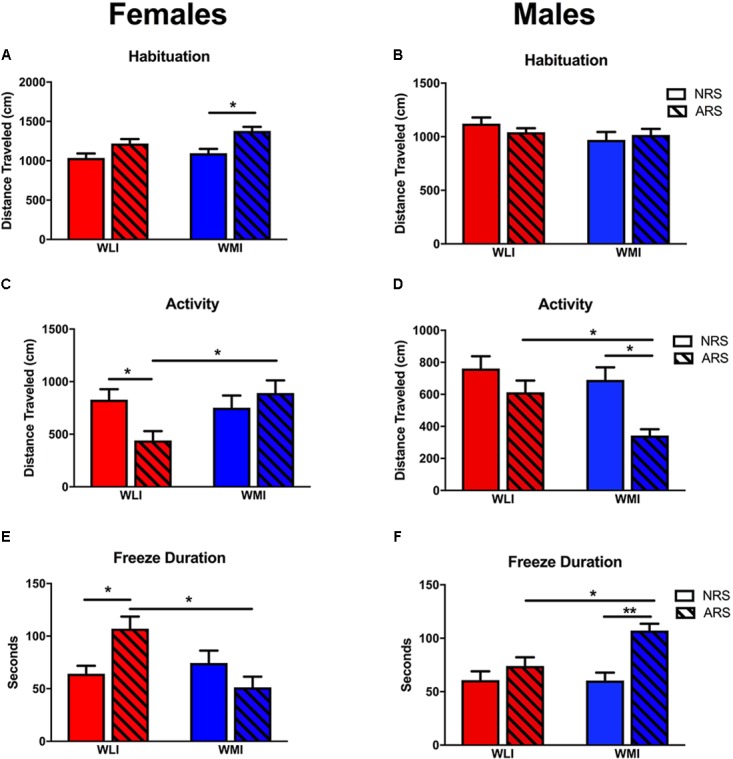
Fear Memory in CFC. Pre-CFC activity, measured by distance traveled in females **(A)** and males **(B)**. Distance traveled prior to the conditioning stimulus did not differ significantly between strains or acute stress (ARS) and no-stress (NRS) conditions except for WMI females, which showed increased activity after acute restraint stress. Distance traveled on the second day of the contextual fear conditioning did not differ between NRS WLI and WMI females **(C)** or NRS males **(D)**. However, WLI females and WMI males showed significantly decreased distance traveled after ARS. Showing the inverse relationship, WLI females and WMI males showed significantly increased freeze duration after ARS, with no differences in fear duration between the strains in the NRS condition **(E**,**F)**. Values are shown as mean ± SEM; ^∗^*p* < 0.05, ^∗∗^*p* < 0.01, *post-hoc* following ANOVA. WLI NRS male *n* = 26; NRS female *n* = 23; AS male *n* = 17, AS female *n* = 13; WMI NRS male *n* = 15, NRS female *n* = 18, AS male *n* = 15, AS female *n* = 10.

During the fear learning phase of CFC (Day 1), the activity of the rats and the responsiveness to the foot-shocks were measured by the distance traveled prior to the shock. In general, males were more active than females [sex, *F*(1,117) = 11.15, *p* < 0.001] (Figures 2A,B). Although there were no strain differences in distance traveled during habituation, WLI males and WMI females were in general more active than the sex-matched opposite strain [strain × sex, *F*(1,117) = 5.16, *p* < 0.05]. Exposure to acute restraint stress 48 h prior to the CFC test significantly increased travel distance during the habituation period [stress, *F*(1,117) = 6.24, *p* = 0.01], specifically in females but not in males [stress × sex, *F*(1,117) = 8.26, *p* < 0.01].

#### Fear Memory

When exposed to the CFC chamber a second time without the shock, both males and females showed a significant difference in activity after stress [stress: *F*(1,129) = 7.55, *p* < 0.01]. There was a significant strain-by-sex-by-stress interaction for activity [*F*(1,129) = 7.23, *p* < 0.01). Previously stressed ARS WLI females showed attenuated activity compared to non-stressed NRS WLI females (Figure 2C). The opposite pattern was observed in males where ARS WMI males demonstrated attenuated activity compared to NRS WMI males (Figure 2D). No attenuation in activity was observed for ARS WMI females or ARS WLI males relative to their respective NRS controls.

The inverse, but also significant, change was seen in freeze duration between the NRS and ARS males and females [stress: *F*(1,129) = 8.79, *p* < 0.01]. Freeze duration after stress were significantly and sex-dependently different between WLIs and WMIs. Increased freeze duration relative to NRS control rats was seen for ARS WLI females, but not in ARS WMI females (Figure 2E). Similarly, ARS WMI males showed increased freeze duration, an indication of enhanced fear memory, compared to NRS WMI males [Figure 2F, strain × sex, *F*(1,129) = 8.51, *p* < 0.01; strain × sex × stress, *F*(1,129) = 13.69, *p* < 0.001].

### Plasma Corticosterone (CORT) and Hippocampal Glucocorticoid (Nr3c1) and Mineralocorticoid (Nr3c2) Receptor mRNA Levels 48 h Post-Acute Restraint Stress

**FIGURE 3:**
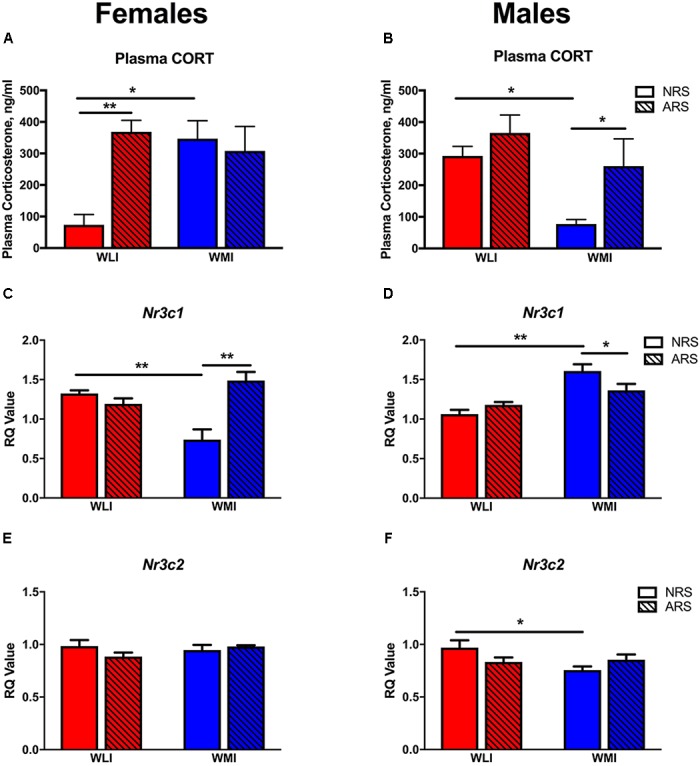
Plasma corticosterone and hippocampal glucocorticoid and mineralcorticoid receptor expression. CORT levels were significantly higher in NRS WMI females **(A)** and NRS WLI males **(B)** compared to the other strain. Acute restraint stress resulted significantly higher CORT levels in the WLI females and WMI males compared to their NRS counterparts **(A**,**B)**, respectively. Hippocampal *Nr3c1* transcript levels showed the opposite pattern to that of plasma CORT. *Nr3c1* expression is decreased in the NRS WMI female **(C)** and in the NRS WLI male **(D)** compared to the other strain in the NRS condition. Acute stress increased *Nr3c1* expression in the WMI female but decreased it in the male hippocampus **(C**,**D)**, respectively. Hippocampal *Nr3c2* transcript levels showed no significant differences in females **(E)**, but decreased expression in NRS WMI compared to NRS WLI **(F)**. Plasma CORT levels, were measured by ELISA, in samples collected 48 h after the restrain stress or no stress applied. Transcript levels were measured in the hippocampus collected at the same time. The RT-PCR used GAPDH as the housekeeping gene and a general calibrator. Relative quantification (RQ) employed the 2^-ΔΔCt^ method. Values are shown as mean ± SEM; ^∗^*p* < 0.05; ^∗∗^*p* < 0.01. NRS, no stress; ARS, acute restraint stress; CORT, corticosterone; *Nr3c1*, glucocorticoid receptor; *Nr3c2*, mineralcorticoid receptor. WLI NRS male *n* = 5(CORT), 4(*Nr3c1*), 6(*Nr3c2*); NRS female *n* = 6(CORT), 5(*Nr3c1*), 8(*Nr3c2*); ARS male *n* = 4(CORT), 5(*Nr3c1*), 5(*Nr3c2*); ARS female *n* = 6(CORT), 6(*Nr3c1*), 6(*Nr3c2*); WMI NRS male *n* = 5(CORT), 4(*Nr3c1*), 6(*Nr3c2*); NRS female *n* = 4(CORT), 4(*Nr3c1*), 8(*Nr3c2*); ARS male *n* = 4(CORT), 5(*Nr3c1*), 5(*Nr3c2*); ARS female *n* = 6(CORT), 6(*Nr3c1*), 6(*Nr3c2*).

Plasma CORT levels were measured in both the NRS and ARS groups at 48 h after the ARS group received restraint stress. CORT levels differed by strain and sex. Specifically in females, baseline (NRS) CORT levels were significantly higher in the WMIs compared to the WLIs, while the opposite was true in males [strain × sex, *F*(1,32) = 12.31; *p* < 0.01; Figures 3A,B]. Plasma CORT levels after stress were generally higher than in the NRS group [stress, *F*(1,32) = 11.30; *p* < 0.01], but they significantly and sex-dependently differed between WLIs and WMIs [strain × sex × stress, *F*(1,32) = 10.54, *p* < 0.01]. ARS significantly increased CORT levels in WLI females but did not result in a further increase in CORT levels in WMI females (Figure 3A). In direct contrast to the results in females, ARS significantly increased CORT levels of the WMI males only, with no further increase in CORT levels in WLI males (Figure 3B).

Hippocampal *Nr3c1* transcript levels, measured in the same animals as plasma CORT, did not show overall significant sex or strain differences although the trend (*p* < 0.1) was there for both variables [strain, *F*(1,31) = 3.46; *p* = 0.072; sex, *F*(1,31) = 3.94; *p* = 0.056; Figures 3C,D). However, there were significant strain-by-sex interactions [strain × sex, *F*(1,31) = 18.60; *p* < 0.001]. In females, *Nr3c1* mRNA levels were significantly higher in the WLI hippocampus, while in males, expression was greater in the WMI hippocampus without prior restraint stress compared to the other strain. However, in contrast to the ARS-induced changes in plasma CORT, acute restraint stress increased hippocampal *Nr3c1* expression in the WMI female, but decreased it in the WMI male hippocampus [stress, *F*(1,31) = 4.30; *p* < 0.05; strain × stress, *F*(1,11) = 4.85; *p* < 0.05; sex × stress, *F*(1,31) = 9.97, *p* < 0.01; strain × sex × stress, *F*(1,31) = 27.71, *p* < 0.001].

Hippocampal *Nr3c2* expression was in general higher in females than in males [sex, *F*(1,42) = 7.29, *p* = 0.01; Figures 3E,F]. The prior stress-induced changes were small and strain dependent [strain × stress, *F*(1,42) = 6.76, *p* = 0.013].

**FIGURE 4:**
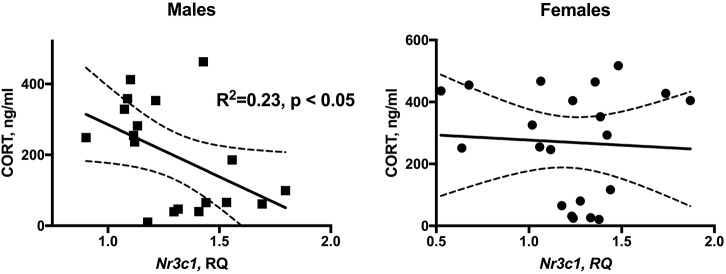
Significant inverse relationship between plasma CORT levels and hippocampal *Nr3c1* expression in male WLI and WMI rats. There is no relationship in females. NRS and ARS data were combined. *n* = 18 (males) and *n* = 20 (females).

The overall association between plasma CORT levels and hippocampal *Nr3c1* expression is shown on Figure 4. In males, linear regression identified a significant negative association between plasma CORT and hippocampal *Nr3c1* transcript levels [*F*(1,16) = 4.78, *p* < 0.05]. No association was detected for females.

### Operant Oral Alcohol Self-Administration

**FIGURE 5:**
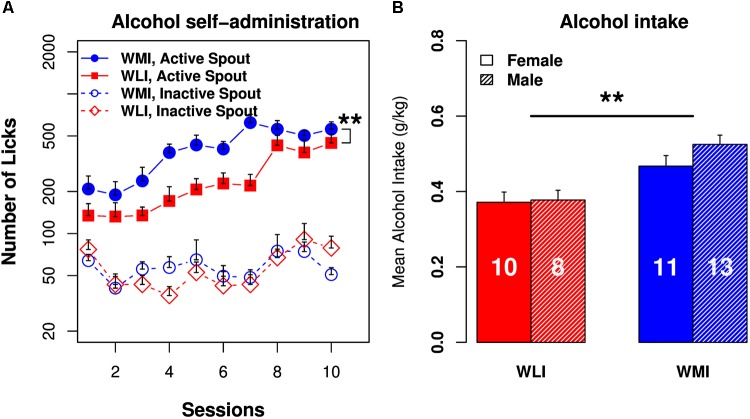
Operant licking alcohol self-administration in WLI and WMI rats. **(A)** Both WMI and WLI rats licked more on the active spouts that delivered EtOH (6.5%) than on the inactive spouts. WMI licked significantly more than the WLI on the active spouts. The number of licks on the inactive spouts were not significantly different between strains. **(B)** Average alcohol intake per session was significantly greater in the WMI than in the WLI strain of animals. There was no sex difference in alcohol intake. Sample sizes are shown in the bar. ^∗∗^*p* < 0.01.

The number of licks on the active spout by the WMI rats were greater than those of the WLI rats throughout the 10 daily test sessions (Figure 5A). Repeated measures ANOVA found a significant strain effect [*F*(1,30) = 9.01, *p* = 0.005]. The differences in licks on the inactive spouts were not significantly different between the strains [*F*(1,30) = 0.007, *p* > 0.05]. WLIs licked 134.5 ± 29.1 times on the active spout and 76.9 ± 13.1 times on the inactive spout during the first session. WMIs licked 208.5 ± 49.9 times on the active spout and 63.9 ± 7.1 times on the inactive spouts during the first session. The number of licks on the active spout increased significantly across the test sessions [*F*(9,143) = 6.9, *p* = 2.68 × 10^-8^) in both strains. By session 10, WLI licked 446.0 ± 67.8 times on the active spout and 78.8 ± 16.8 times on the inactive spout. The number of licks on the active spouts were significantly greater than those on the inactive spouts [*F*(1,11) = 89.4, *p* = 1.29 × 10^-6^) in both strains. Increases in the number of active licks across the test sessions was statistically highly significant [*F*(1,19) = 133.5, *p* = 4.9 × 10^-10^). For the WMIs, the number of licks increased to 558.9 ± 71.4 on the active spout but declined to 50.8 ± 6.2 on the inactive spout in session 10 (Figure 5A).

Alcohol intake (mg/kg bodyweight; Figure 5B) did not change significantly across the sessions in the WLI strain [*F*(9,123) = 1.35, *p* > 0.05] but increased significantly in the WMI strain [*F*(9,175) = 2.86, *p* < 0.01]. The amount of alcohol consumed was independent of sex for both strains [*F*(1,6) = 5.16, *p* > 0.05 for WLI and *F*(1,15) = 1.18, *p* > 0.05 for WMI]. When the two strains were compared, alcohol intake in the WMI was significantly greater than that of the WLI [*F*(1,21) = 7.6, *p* < 0.01].

### Heritability

Heritability can be estimated from inbred strain trait data and used to determine the feasibility of genetic mapping. Heritabilities of 0.2 or higher demonstrate that genetic factors are a major contributor to phenotypic variation. Broad sense heritability or *H*2 (Hegmann and Possidente, 1981; Falconer and Mackay, 1996) was estimated from ANOVA for SEFL and alcohol intake by dividing the variance component among strains [AS_com_, calculated as the mean square between strain subtracted by the variance component within strains (WS_com_, mean square within strain)] by the total variance component (AS_com_ + WS_com_). Heritability of the SEFL was estimated at 0.74 and 0.70 for females and males, respectively. The effect size (Cohen’s D) for SEFL is 1.53 and 1.13 for females and males, respectively. Both are large effects. Heritability for alcohol self-administration was 0.45 with no sex difference. The corresponding effect size is 0.76 (medium size). These heritability estimates indicate that a significant proportion of variation in these phenotypes is due to genetic factors.

## Discussion

To our knowledge, this is the first study showing both *intrinsically* higher alcohol consumption and SEFL in an animal model. The major findings of the study include moderately high heritability and sex and strain differences in SEFL and high heritability and sex-independent strain differences in alcohol consumption. We demonstrated increased alcohol consumption of the WMI strain, developed as a genetic model of depression-like behavior, compared to its genetically close control WLI strain. We also confirmed previously observed sex differences in SEFL behavior, namely that male WMI demonstrate SEFL behavior (decreased activity and increased fear memory), while female WMI do not. Even more intriguing is that the control WLI strain displayed an inverse effect, with the WLI females exhibiting SEFL, while male WLIs did not. These behavioral differences in stress response coincide with the sexually dimorphic hormonal response to stress between the WLIs and WMIs; differences in NRS and ARS plasma CORT levels paralleled the presence of SEFL in contextual fear conditioning with higher CORT levels associated with ARS in female WLI and male WMI only.

Alcohol self-administration was conducted without sucrose fading or water deprivation. Both of these strategies could affect the motivation to obtain alcohol that may or may not be relevant to increased alcohol intake. In the present study, the voluntary alcohol consumption of WMIs was significantly greater compared to those of WLIs, with strain having a medium effect size on alcohol drinking. This finding indicates a genetic predisposition, similarly to those occurring in many subjects with AUD (Foulds et al., 2015). Although the amount of alcohol consumed by these strains in the 1 h test period is only ∼0.5 g/kg body weight, it is similar to consuming two standard alcoholic drinks by a 70 kg human. Future experiments will seek to increase alcohol consumption by extending the length of the alcohol sessions and/or force withdrawal periods between sessions, which has been shown to better model human alcohol drinking (Smutek et al., 2014) The parental WKY strain of both the WMI and the WLI has been accepted as an animal model of depression (Pare and Redei, 1993a; Dugovic et al., 2000; Jeannotte et al., 2009; Tizabi et al., 2012). The WKY strain is also reported to consume more alcohol than the Sprague-Dawley strain (Pare et al., 1999) or the Wistar strain (Jiao et al., 2006; Yaroslavsky and Tejani-Butt, 2010). Others have reported that WKYs consume low levels of alcohol (Li and Lumeng, 1984; Khanna et al., 1990) compared to other strains, albeit in a sex-specific manner. These latter studies employ two different WKY strains, both of which are different from our parental WKY strain and from those used in the studies showing elevated alcohol consumption compared to other strains. This is a significant issue as we and others have reported genetic and phenotypic sub-strain differences according to suppliers of the WKY strain of animals (Kurtz and Morris, 1987; Kurtz et al., 1989; Matsumoto et al., 1991; Deschepper et al., 1997; Pare and Kluczynski, 1997; Okuda et al., 2002; Will et al., 2003; Zhang-James et al., 2013).

Strain and sex-dependent SEFL results presented in this study both confirmed and extended previous findings. This model was developed originally as an animal model of acute stress that parallels many symptoms of PTSD (Perusini et al., 2016). The SEFL paradigm developed by Fanselow and colleagues (Rau et al., 2005), employs a series of 15 shocks, which leads to subsequently exaggerated contextual fear. We have generated the same SEFL using a single 2 h acute restraint stress 48 h prior to the commencement of the contextual fear conditioning, similarly to previous studies (Cordero et al., 2003; Rodriguez Manzanares et al., 2005; Blouin et al., 2016). The similarity between the present and previous studies is the profound sex difference, namely that WMI males show SEFL, an indicator of enhanced fear memory, while WMI females do not. However, a surprising finding is the opposite effect in WLI females, where WLI females show SEFL, while WLI males do not.

We assessed the possibility that these differences are related to the basic anxiety-like behavior in these strains. The paternal WKY strain shows anxiety-like behavior in multiple behavioral paradigms (Pare and Redei, 1993a,b; Pare, 1994; Tizabi et al., 2010). Although the selective breeding of WKYs into the WLI and WMI strains was based on depression-like behavior in the forced swim test, the segregation of the depression- and anxiety-like behaviors of the male WMIs/WLIs occurred progressively throughout the generations (Mehta et al., 2013). Thus, male WMIs show lower levels of anxiety than WLIs, while both WLI and WMI females show equal level of anxiety-like behavior. If the higher trait anxiety is the cause of these paradoxical SEFL responses, then WLI males and both WLI and WMI females should show greater fear memory than that of WMI male. Because that is not the case, it is unlikely that the differences in SEFL are a result of differences in trait anxiety.

However, the stress-state of these animals differed significantly, as measured by plasma CORT levels at the time when contextual fear learning would be initiated. The mechanism of the unexpected findings; elevated CORT levels in the unstressed WMI females and WLI males and persistent plasma CORT elevations in the WLI females and WMI males 48 h after an acute restraint stress will need to be explored in the future. The high unstressed CORT levels of WMI females are not likely related to the depression-like behavior in these strains, as we have shown previously that the depression-like behavior of the parental WKY strain and CORT regulation are independent traits (Solberg et al., 2003), and the WLI male also show these high CORT levels. Sensitization of the hypothalamic-pituitary- by prior exposure to a single immobilization session has been shown to lasts for around 1 week (Belda et al., 2008, 2012, 2015). Whether the removal from the home cage and euthanasia is the cause, or whether the time between the removal from home cage and the collection of blood samples is sufficient time to show this presumed sensitization will need to be determined. The lack of change by ARS in plasma CORT levels of WLI males and WMI females might be related to their already high unstimulated levels of plasma CORT. Acute stress could possibly increase CORT levels in these animals as well in the usual stress response time frame such as 20–40 min after the onset of acute stress.

The surprising parallel between the plasma CORT level differences between non-stressed and stressed males and females and their SEFL measures suggest that a differential stress regulation drives the strain-, and sex-specificity of SEFL. In contextual fear conditioning, the presence of CORT is needed prior to conditioning to support the consolidation of a long-term representation of the context (Pugh et al., 1997). Administration of CORT is known to enhance fear conditioning in rats (Cordero et al., 2003; Thompson et al., 2004; Perusini et al., 2016). Inversely, blocking CORT synthesis prior to stress, but not any time after that, attenuates SEFL (Perusini et al., 2016). In that study it is concluded that CORT is necessary, but not sufficient to SEFL. In the present study, we observed large differences in the non-stressed CORT levels between the strains, but there were no differences in the stressed CORT levels 48 h post-stress. Thus, persistent elevation of plasma CORT levels without stress are not sufficient to elicit enhanced fear responses, but rather the elevation of levels from normally low basal CORT levels in response to stress are necessary. It is important to note, that patients with PTSD are often show lower cortisol levels compared with individuals without PTSD (Mason et al., 1986; Yehuda, 1997, 2001). Lower basal CORT levels in the WMI males and WLI females may be the necessary and sufficient criteria for showing SEFL. This is an intriguing possibility that needs to be explored further.

The inverse relationship between plasma CORT and hippocampal glucocorticoid receptor expression is further evidence of the sex-specific differential regulation of the stress response in these strains. The linear regression between plasma CORT levels and hippocampal *Nr3c1* expression clearly shows that males have a significant association between these parameters, while females do not. While female rats are known to have higher basal CORT levels and enhanced CORT responses to stress compared to males, their glucocorticoid-regulated other functions are not enhanced (Hulshof et al., 2012). This implies a reduced efficacy of the CORT in females, including that of regulation of *Nr3c1* expression. As glucocorticoid receptor expression is subject to ligand mediated negative autoregulation, the higher *Nr3c1* expression in the WMI males without stress does suggest downregulation of the hypothalamic-pituitary adrenal axis activity in these animals. Since the WMI males are also the ones showing enhanced fear memory similar to those of subjects with PTSD, the lower basal (no-stress) CORT levels in these animals is in striking agreement with those reported for PTSD patients (Yehuda, 2001). Comparing our findings with that of human PTSD, albeit difficult, resulted in surprising parallels. High GR expression, although not in the hippocampus, but in peripheral blood, predicted high levels of PTSD symptoms in military personnel prior to deployment (van Zuiden et al., 2012; Girgenti and Duman, 2018; Daskalakis et al., 2018). Thus, the high GR expression of WMI males compared to WLI males without prior stress correctly predicts their SEFL, just as the opposite strain differences in GR expression predict it in females.

In the present study no plasma CORT levels were measured after the alcohol intake experiment. Although it has been suggested that individual variation in alcohol intake may not be related to endogenous CORT levels (Fahlke et al., 1994), whether that is true in the case of the WLI and WMI strains will need to be determined in the future. Measuring alcohol consumption after SEFL or conducting the SEFL after alcohol dependence could also answer important questions about this animal model in the future.

In conclusion, the male WMI is a unique, genetic model of comorbidity of PTSD- and AUD-like traits as it differs in these phenotypes from its nearly isogenic WLI control strain. This rodent model provides a unique opportunity to disentangle these comorbid phenotypes and dissect the influences of genetic and sex-specific factors, as the WMI strain likely harbor genetic variation(s) predisposing them to both higher alcohol intake and increased susceptibility to exaggerated fear learning following stress exposure.

## Author Contributions

HC, MM, and ER designed the study. PL, GS, TW, STJ, and HC carried out the experimental work. PL, GS, ER, and HC analyzed the data. PL, MM, ER, and HC drafted the manuscript. All authors approved the final manuscript.

## Conflict of Interest Statement

The authors declare that the research was conducted in the absence of any commercial or financial relationships that could be construed as a potential conflict of interest.
