# Supplementary material for: Genetic Model to Study the Co-Morbid Phenotypes of Increased Alcohol Intake and Prior Stress-Induced Enhanced Fear Memory
Source: Front Genet. 2018 Nov 27;9:566. doi: 10.3389/fgene.2018.00566 (PMC6277590; doi:10.3389/fgene.2018.00566)
Supplement: Supplementary file 1 [file Table_1.DOCX]

| Supplemental Table 1. Quantitative RT-PCR primer sequences. | |
| --- | --- |
| **Gene** | **Sequence 5’ - 3’** |
| *Nr3c1* | F: AACAGACTTTCGGCTTCTGGAA  R: TGGAACGCTGGTCGACCTAT |
| *Nr3c2* | F: TGGAAGGGCAACACAACTATCTG  R: AGTTCTTTCGCCGAATCTTATCA |
| *Gapdh* | F: CAACTCCCTCAAGATTGTCAGCAA  R: GGCATGGACTGTGGTCATGA |

**Supp. Table 1.** Abbreviations: F, forward; R, reverse.
